# Trends in Use of and Medicare Spending on Short-Course Radiotherapy for Lymphomas From 2015 to 2019

**DOI:** 10.1001/jamahealthforum.2022.1815

**Published:** 2022-07-01

**Authors:** Kathryn R. Tringale, Harper Hubbeling, Fumiko Chino, Carla Hajj, Joachim Yahalom, Brandon S. Imber

**Affiliations:** 1 Department of Radiation Oncology, Memorial Sloan Kettering Cancer Center, New York, New York

## Abstract

This cross-sectional study uses Centers for Medicare & Medicaid Services payment data to examine use of short-course radiotherapy from 2015 to 2019 among Medicare beneficiaries with indolent lymphoma.

## Introduction

With increasing medical costs and concerns about COVID-19 exposure, reducing financial and time burdens of cancer treatment is critical.^1^ In radiation oncology, use of hypofractionated short-course radiotherapy (SC-RT) can decrease burden on patients and the health care system.^2,3^ For indolent lymphomas, existing guidelines recommend 24 Gy in 12 treatments; however, recent experience has shown shorter regimens (ie, 4 Gy in 1-2 treatments) are effective for palliation and possibly cure.^4,5^ Short-course RT is associated with equivalent overall survival and offers logistical and toxicity benefits with modestly inferior local control.^4^ Emergency radiotherapy guidelines advocated SC-RT to reduce COVID-19 exposure.^1^ Extent of adoption and financial implications of SC-RT before COVID-19 remain unclear. We evaluated SC-RT use with US episode-based payment data from the Centers for Medicare & Medicaid Services.^6^

## Methods

This cross-sectional study was approved by Memorial Sloan Kettering Cancer Center’s institutional review board; informed consent was waived because data were publicly available. The study followed the STROBE reporting guideline. Radiotherapy episodes from 2015 to 2019 were analyzed for Medicare beneficiaries 65 years or older who did not receive systemic therapy and lived more than 90 days after RT for lymphoma (eMethods in the Supplement). Delivery technique (conventional vs intensity-modulated RT [IMRT]), treatment year (2015-2019), age (65-74, 75-84, or ≥85 years), and site of care (freestanding vs hospital affiliated) were covariables. Use of SC-RT (1-10 treatments) vs long-course RT (LC-RT; 11-20 treatments) was evaluated by multivariable logistic regression, with the interaction between site of care and year investigating longitudinal patterns. Medicare spending was evaluated with multivariable linear regression. Data were analyzed using SAS Enterprise Guide, version 7.1. Two-sided *P* = .05 was significant.

## Results

Of 10 447 radiation episodes, 50% were among women and 81% were among patients younger than 85 years. Most (78%) used conventional RT; however, IMRT use increased from 17% in 2015 to 25% in 2019 (*P* < .001). Most patients (71%) received LC-RT. Receipt of SC-RT was associated with older age (≥85 vs 65-74 years: odds ratio [OR], 2.18; 95% CI, 1.94-2.45), hospital-affiliated vs free-standing site of care (OR, 1.74; 95% CI, 1.57-1.93), and conventional RT vs IMRT (OR, 5.18; 95% CI, 4.45-6.02) (Table). Increased SC-RT use was unique to hospital-affiliated sites (Figure).

**Table.** Results From Multivariable Logistic Regression Assessing Use of SC-RT and From Multivariable Linear Regression Modeling of Medicare Spending

Variable	Use of SC-RT		Total spending		Professional spending		Technical spending	
	Adjusted OR (95% CI)^a^	*P* value	β (95% CI), $^b^	*P* value	β (95% CI), $^b^	*P* value	β (95% CI), $^b^	*P* value
Age, y								
65-74	1 [Reference]	NA	1 [Reference]	NA	1 [Reference]	NA	1 [Reference]	NA
75-84	1.27 (1.15 to 1.40)	<.001	62 (−25 to 149)	.16	18 (−2 to 39)	.08	44 (−27 to 114)	.22
≥85	2.18 (1.94 to 2.45)	<.001	212 (104 to 320)	<.001	42 (16 to 68)	.001	169 (82 to 257)	<.001
Year	1.13 (1.10 to 1.17)	<.001	−59 (−86 to −31)	<.001	−20 (−27 to −13)	<.001	−39 (−61 to −16)	<.001
Technique								
IMRT	1 [Reference]	NA	1 [Reference]	NA	1 [Reference]	NA	1 [Reference]	NA
Conventional RT	5.18 (4.45 to 6.02)	<.001	–5167 (–5264 to –5070)	<.001	–719 (–743 to –696)	<.001	–4448 (–4526 to –4369)	<.001
Site of care								
Freestanding	1 [Reference]	NA	1 [Reference]	NA	1 [Reference]	NA	1 [Reference]	NA
Hospital affiliated	1.74 (1.57 to 1.93)	<.001	298 (213 to 383)	<.001	−87 (−107 to −66)	<.001	385 (316 to 454)	<.001
Regimen								
SC-RT	NA	NA	1 [Reference]	NA	1 [Reference]	NA	1 [Reference]	NA
LC-RT	NA	NA	3740 (3650 to 3830)	<.001	572 (550 to 594)	<.001	3168 (3096 to 3241)	<.001

Abbreviations: IMRT, intensity-modulated radiotherapy; LC-RT, long-course radiotherapy; NA, not applicable; OR, odds ratio; SC-RT, short-course radiotherapy.

^a^ The ORs are from multivariable logistic regression including the covariables listed and the interaction between site of care and year. Use of SC-RT over time was significantly different between freestanding and hospital affiliated centers (Figure).

^b^ Spending was defined as Medicare-reimbursed professional and technical service fees per 90-day RT episode, adjusted to 2019 dollars. Adjusted β coefficients can be interpreted as the difference in mean spending between the groups (or per year when assessing change over time) while holding the other covariables constant.

**Figure.**
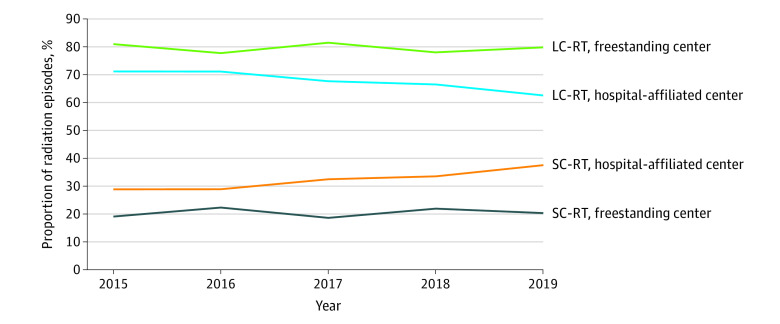
Proportion of Lymphoma Radiation Episodes Delivered With Short-Course (SC) vs Long-Course (LC) Radiotherapy (RT) From 2015 to 2019, Stratified by Site of Care Short-course RT involves 1 to 10 treatments and LC-RT, 11 to 20 treatments. The interaction between site of care and year of treatment was significant (*P* = .01) when controlling for patient age and RT technique (conventional RT vs intensity-modulated RT) on multivariable logistic regression.

Median total spending for SC-RT was $4278 (IQR, $2962-$5569) vs $8484 (IQR, $6919-$11 330) for LC-RT. For SC-RT, median total spending was $8048 (IQR, $5404-$9403) with IMRT vs $4121 (IQR, $2876-$5310) with conventional RT. Median total LC-RT spending was $13 085 (IQR, $11 445-$14 842) with IMRT vs $7657 (IQR, $6422-$8992) with conventional RT. Professional services–related spending was reduced with SC-RT (adjusted β, $572; 95% CI, $550-$594; *P* < .001), conventional RT, younger patients, and hospital-affiliated sites (Table). Technical services–related spending was reduced with SC-RT (adjusted β, $3168; 95% CI, $3096-$3241; *P* < .001), younger patients, conventional RT, and freestanding sites (Table). In linear regression, delivery technique was associated with the largest contribution to total spending, followed by course length.

## Discussion

Most Medicare beneficiaries treated with radiation monotherapy for lymphoma between 2015 and 2019 received LC-RT. Use of LC-RT has important financial implications given higher total spending. Despite lower health care spending and reduced time, travel, and costs for patients, SC-RT was used in only 29% of patients by 2019. Whereas practice patterns may reflect site-specific case mixes, ensuring differences do not reflect competing financial incentives owing to reimbursement structures or opinions on evidence-based practice is important. Increases in spending were greatest for IMRT; therefore, regimen and technique should be considered for cost savings. Financial alignment with value-based practice irrespective of site of care is critical; future studies may help refine the balance between local control, patient treatment-related burden, and health care spending. Wider adoption of SC-RT may help reduce systemwide costs and optimize personalized RT decision-making. Limitations include lack of available clinical variables; despite efforts to limit the analysis to indolent lymphomas for which SC-RT is considered, more aggressive subtypes may have been included.

## Notes

Supplement.
eMethods
