# Supplementary material for: Trends in Use of and Medicare Spending on Short-Course Radiotherapy for Lymphomas From 2015 to 2019
Source: JAMA Health Forum. 2022 Jul 1;3(7):e221815. doi: 10.1001/jamahealthforum.2022.1815 (PMC9250056; doi:10.1001/jamahealthforum.2022.1815)
Supplement: Supplement. — eMethods [file jamahealthforum-e221815-s001.pdf]

Supplementary Online Content

Tringale KR, Hubbeling H, Chino F, Hajj C, Yahalom J, Imber BS. Trends in use of and Medicare spending on short-course radiotherapy for lymphomas from 2015 to 2019. *JAMA Health Forum*. 2022;3(7):e221815. doi:10.1001/jamahealthforum.2022.1815

eMethods

This supplementary material has been provided by the authors to give readers additional information about their work.

© 2022 Tringale KR et al. *JAMA Health Forum*.

eMethods

Two separate datasets were available per the RO-APM website (2015-2017 and 2017-2019), therefore the 2017 data was included from the more recent dataset and the two datasets were combined to form one comprehensive dataset for the 5-year period of 2015-2019. Episodes were selected for patients who received radiation therapy for lymphoma (ICD10 codes C81-C86, C88, C91.4). Younger patients (<65 years of age) were excluded to have a clean cohort of Medicare beneficiaries excluding those who have Medicare for special considerations. Episodes for radiation therapy delivered with protons or brachytherapy, those with more than 20 fractions, and those that were delivered with multiple techniques (e.g., both conventional external beam and intensity-modulated radiation therapy) were excluded since these are not the standard approaches expected for treatment of indolent lymphoma. Given the lack of data on tumor histology, patients who received chemotherapy or who died within 90 days of radiation therapy were excluded as a surrogate marker for more aggressive disease.

© 2022 Tringale KR et al. *JAMA Health Forum*.
